# Age and sex prevalence of infectious dermatoses among primary school children in a rural South-Eastern Nigerian community

**DOI:** 10.11604/pamj.2015.20.182.6069

**Published:** 2015-02-27

**Authors:** Eziyi Iche Kalu, Victoria Wagbatsoma, Ephraim Ogbaini-Emovon, Victor Ugochukwu Nwadike, Chiedozie Kingsley Ojide

**Affiliations:** 1 Department of Medical Microbiology, Federal Medical Centre, Umuahia, Nigeria; 2 Department of Community Health, University of Benin, Nigeria; 3 Institute of Lassa Fever Research and Control, Irrua Specialist Teaching Hospital, Irrua, Nigeria; 4 Department of Medical Microbiology, Federal Medical Centre, Abeokuta, Nigeria; 5 Department of Medical Microbiology, Federal Teaching Hospital, Abakaliki, Nigeria

**Keywords:** Prevalence, infectious dermatoses, skin, children, primary schools

## Abstract

**Introduction:**

Various dermatoses, due to their morbidity characteristics, have been shown to negatively impact on learning. The most epidemiologically important seem to be the infectious types because of their transmissibility and amenability to simple school-health measures. The aim of this study was to assess the prevalence and sex/age correlates of infectious dermatoses in a rural South-eastern Nigerian community.

**Methods:**

The pupils were proportionately recruited from the three primary schools based on school population. Stratified simple random sampling method was adopted and a table of random numbers was used to select required pupils from each arm. Clinical and laboratory examination was done to establish diagnoses of infectious skin disease. Data collected were analyzed using SPSS version 16.

**Results:**

The 400 pupils consisted of 153 males and 247 females. Age range was between 6 and 12 years. The prevalence of infectious dermatoses was 72.3%. The five most prevalent clinical forms of infectious dermatoses, in order of decreasing prevalence, were tinea capitis (35.2%), scabies (10.5%), tinea corporis (5.8%), tinea pedis (5.5%), and impetigo (5.0%). More cases, generally, occurred among males than females (80.4% vs 67.2%)); while some specific clinical types, pediculosis and seborrheic dermatitis, exhibited predilection for females. Pyodermas and scabies were significantly more prevalent in the 7-9 age-group; while tinea capitis, tinea corporis, seborrheic dermatitis and pediculosis were more associated with ≥10 age-group.

**Conclusion:**

Infectious dermatoses were highly prevalent in the surveyed population. Many of the clinical types exhibited sex- and age-specificity.

## Introduction

Infectious dermatoses as used in this study refer to skin diseases that are of presumed bacterial, viral, fungal or parasitic aetiology; and they may be primary or secondary [1, 2]. The primary bacterial dermatoses, also referred to as pyodermas, includes impetigo, folliculitis, furunculoses, ecthyma, and cellulitis [1, 3]. The primary viral dermatoses include herpes zoster, herpes simplex, and warts; while the primary fungal dermatoses include pityriasis versicolor, tinea nigra, and dermatophytosis [1, 3, 4]. The main ectoparasitic dermatoses include scabies and pediculosis [1, 3, 4]. Secondary infectious dermatoses include all the superinfections of various aetiopathologies [4]. Examples include eczema herperticum, due to superimposition of herpes simplex lesions on eczematous lesions; and the various secondary pyodermas usually superimposed on atopic dermatitis, dermatophytosis, scabies and papular urticaria lesions in the tropical environment [5–7]. Most of the skin lesions exhibit known typical clinical morphological patterns, along with characteristic sites of predilection [8]. The typical primary school child is aged between six years to twelve years [9]. Statistics indicate that this age group may constitute about 44% of the entire Nigerian population; and up to 60% of this population reside in the rural areas [10]. Children in the primary school age group are not “small adults” [11]. They are yet physically, physiologically and immunologically immature; and so, they are vulnerable to injuries from the environment [11]. Specific characteristics of these children, therefore, include rapid physical and mental development [9]. These result in high nutritional need and rapid development of nutritional deficiencies if they are persistently underfed [12]. Inadequate feeding is, in turn, associated with immunodeficiency and enhanced susceptibility to infection [10]. Furthermore, children in this age group are survivors of the tropical environmental risk factors of high early childhood mortality; and many of these risk factors remain relevant in the primary school age [13]. These risk factors include poverty, male sex, low maternal education, low maternal age, shorter birth intervals, large family size, malnutrition, incomplete immunization and low standards of sanitation [14–16]. The primary school children are also exposed to the typical school hazards: physical injuries, emotional problems and infection [9, 11]. The commonly overcrowded school environment, in developing countries, is a strong dissemination factor as the infectious dermatoses have a high chance of spreading among this group of people who may not have learnt hygiene skills and who tend to be inherently careless about their health [17, 18]. This proneness to infections call for special attention to these children in relation to their health, including their skin health. Furthermore, various dermatoses, due to their morbidity characteristics, have been shown to constitute a serious setback to the education of the child [19, 20]. Although these diseases are not common causes of mortality, they may be common causes of morbidity and may interfere with learning [19, 20]. Among children, the most epidemiologically important of these dermatoses seem to be the infectious types because of their high prevalence and transmissibility [3, 21]. In the study on the prevalence of parasitic skin diseases in Benin, Nigeria, most of the cases were found among children [22]. Infectious dermatoses were the greatest indications of primary health care clinic attendance among children in Enugu, Nigeria, in another study [23]. Reports from several studies in this sub-region show that, due to the physical and socioeconomic environments, the clinical types of most significant prevalence in children include the dermatophytoses, scabies, pediculosis and the pyodermas [24–26]. Fungal dermatoses usually constituted the vast majority of the infectious dermatoses among children in one of the studies [26]. Different authors studying specific infectious skin diseases have found high prevalences of various infectious skin diseases among school children in different parts of Nigeria [27–30]; and several factors, including age and sex, have been shown to be associated [26–30]. There is paucity of data on this subject in eastern Nigeria. The few related studies were hospital-based, and not community-based [24, 23]; and so assessed only the expressed needs, rather than the real needs of the people [25]. As a result of the amenability of these infectious dermatoses to simple public health control efforts, their control can be incorporated into the school health programme, in line with the Nigerian school health policy in 2006 [3, 21]. Adequate epidemiologic database on infectious dermatoses in the reference population is necessary to ascertain the need and mode of interventions. This survey is set to determine the age- and sex-prevalence of dermatoses of infectious origin, among children attending primary schools in Ndi Uduma Awoke community of Ohafia Local Government Area(LGA) of Abia State, Nigeria.

## Methods

**Study area**: Ndi Uduma Awoke, the study area, forms the Uduma Ward of Ohafia (LGA) of Abia State in South-eastern region of Nigeria. An estimate of 25,000 persons reside in this rural community. The primary residents are predominantly subsistence farmers; and have vast arable lands reserved for their shifting cultivation practice. A minority of the primarily resident members of this village are engaged in teaching, artisanship and petty trading as adjunct occupations to farming. Three streams constitute sources of water supply, although a few relatively well-to-do inhabitants get their supplies from water tankers. There is a primary health care centre and two chemist shops that serve the basic health needs of the village. Refuse disposal is by open dumping; while common sites for open excreta disposal are located in the outskirts of the various compounds. There are three registered primary schools in this community, designated A, B and C.

**Study population, design, and duration**: this descriptive cross-sectional study was conducted among primary school pupils in Ndi Uduma Awoke between February, 2012 and February, 2013.

**Advocacy/ethical considerations**: approval was sought and obtained from the Health Research Ethics Committee of University of Benin Teaching Hospital for this research work. After due explanations of the involvements of the project, permissions were obtained from the Head-teachers, the class teachers and the pupils of the surveyed schools. Informed verbal and written consents were obtained from the parents for participation of their children in the study.

**Data collection / sampling methods**: The sample size of 406 was calculated using the prevalence value of 40% [28], and considering possible 10% attrition. All pupils in primaries one to six, between ages 6-12 years, who were present in school and who consented to participate were included; while those pupils in classes below primary one, above 12 years and all who did not consent were excluded. The number of participants from each of the schools (NA, NB, NC) were proportionately determined, with respect to the school populations. Briefly, the population of pupils in schools“A”, “B” and “C”were 440, 360, and 80; making a total of 880 primary school children in the community. Thus, the NA of 203 pupils was derived by multiplying the sampling fraction (406/880 or 0.461) by 440; NB of 166 was the product of the sampling fraction and 360; while NC of 37 was the product of the sampling fraction and 80. School A was stratified into six classes with known class populations (P1, P2, P3, P4, P5, P6) of 59, 83, 71, 59, 97, 71 respectively. By multiplying each respective class population by the sampling fraction (0.461), the number of participants from each class (N1, N2, N3, N4, N5 and N6) of school “A”were found to be 27, 38, 33, 27, 45, 33 respectively. In school “B”, the class populations (P1, P2, P3, P4, P5 and P6) were 63,59,53,53,67, and 55 respectively; and N1, N2, N3, N4, N5 and N6 were 30, 28, 25, 25, 32, and 26 respectively, making the total NB of 166. Similarly, school “C”was stratified into six classes and the allocated NC of 38 was proportionately distributed among the 6 classes in accordance with the populations of each class. Simple random sampling technique, using a table of random numbers, was adopted in selection of the 406 samples from the arms of each class. However, six (6) of them did not participate. Data was, thus, collected from 400 pupils only.

**Training of field assistants**: seventeen field assistants, made of three nurses and fourteen teachers, were trained to assist with the administration of the questionnaires and other aspects of data collection logistics. The teachers were all staff of the primary schools in Ndi Uduma Awoke. A primary school in a neighboring community was used as pilot study area.

**Tool for data collection**: I. Pre-tested structured, interviewer-administered questionnaire. Age-group classification was according to Uneke [27]. II. Clinical diagnosis: history, physical examination, with the aid of magnifying glass and self-instructional manuals [31]. III. Laboratory diagnosis. Some atypical lesions suspected to be cutaneous mycoses or pyodermas were confirmed by direct microscopy of scrapings from the lesions, in accordance with standard procedures [32]. Scrapings from some suspected scabies lesions were also examined microscopically for mites, their scybala and their eggs. Products of wet combing were also examined both macroscopically and microscopically for knits. IV. Photograph of lesions. Snapshots of the lesions, including the non-specific ones, were taken for possible review by a consultant dermatologist. (APPENDIX) SAMSUNG S630 6.0 Mega-Pixel Digital Camera was used. V. Aetiological classification was essentially based typical morphologies of the lesions, and not on isolation of the pathogens. For the purpose of this study, seborrheic dermatitis lesions were classified as fungal dermatoses.

**Analysis of data**: data entry and analysis utilized SPSS version 16 spread sheet and software. Chi2 and Fisher's Exact tests were used to assess significance of associations. Significance of associations were based on P-values < 5%.

## Results

Four Hundred and Six of the pupils were selected to participate in the study but only 400/406 consented and actually participated, giving a response rate of 98.5%.

**Sex and age characteristics of the study population**: one hundred and fifty three (38.3%) of the pupils were males; while 247 (61.7%) of them were females. This gave a male: female ratio of 1: 1.5 (Table 1). The ages of the pupils ranged from 6 to 12 years; with a mean age of 9.43 ± 2.35 years (Table 2). One hundred and four (26%) of the pupils were aged 6 to 7 years; 83 (21%) were aged 8 to 9 years; 144 (36%) were aged 10 to 11; while 68 (17%) were 12 years in age. The mean age of the males was 9.6 ± 2.1; while the mean age of the females was 8.6 ± 1.8.

**Table 1:** Prevalence of specific clinical forms by sex

**Tinea capitis**					
**Sex**	**positive (%)**	**negative (%)**	**total (n = 400)**	**x^2^**	**p-value**
Males	77(50.3)	76 (49.7)	**153**	**55.33**	0.0001
Females	34 (13.8)	213 (86.2)	**247**	**-**	-
**Tinea corporis**					
Sex	**positive (%)**	**negative (%)**	**total (n = 400)**	**x^2^**	**p-value**
Males	9(5.9)	144(94.1)	**153**	**0.008**	0.533
Females	14(5.7)	86(94.3)	**247**	**-**	**-**
**Pityriasis versicolor**					
Sex	**positive (%)**	**negative (%)**	**total (n = 400)**	**x^2^**	**p-value**
Males	0(0.0)	153(100.0)	**153**	**5.06**	0.024
Females	8 (3.2)	239 (96.8)	**247**	**-**	**-**
**Athlete's foot**					
Sex	**positive (%)**	**negative (%)**	**total (n = 400)**	**x^2^**	**p-value**
Males	5(3.3)	148	**153**	**0.006**	0.535
Females	17(3.2)	230	**247**	**-**	**-**
**Pyodermas**					
Sex	**positive (%)**	**negative (%)**	**total (n = 400)**	**x^2^**	**p-value**
Males	20 (13.1)	133 (86.9)	**153**	**3.763**	0.063
Females	28 (3.2)	219 (96.8)	**247**	**-**	**-**
**Pediculosis**					
Sex	**positive (%)**	**negative (%)**	**total (n = 400)**	**x^2^**	**p-value**
Males	0(0.0)	153 (100.0)	**153**	**5.06**	0.024
Females	8 (3.2)	239 (96.8)	**247**	**-**	**-**
**Scabies**					
Sex	**positive (%)**	**negative (%)**	**total (n = 400)**	**x^2^**	**p-value**
Males	22 (14.4)	131(85.6)	**153**	**0.006**	0.614
Females	20 (9.4)	227(90.6)	**247**	**-**	**-**

**Table 2:** Prevalence of specific clinical types by age-groups

**Tinea capitis**					
**Age-groups**	**positive (%)**	**negative (%)**	**total n = 400**	**x** ^**2**^	**p-value**
6-9 years	56 (29.8)	132(70.2)	**188**	4.64	0.041
≥ 10 years	85(40.1)	127(59.9)	**212**		
**Tinea corporis**					
Age-groups	**positive (%)**	**negative (%)**	**total n = 400**	**x** ^**2**^	**p-value**
6-9 years	0 (0.0)	188 (100.0)	**188**		
≥ 10 years	23 (10.8)	189(89.2)	**212**		
**Pityriasis versicolor**					
Age-groups	**positive (%)**	**negative (%)**	**total n = 400**	**x** ^**2**^	**p-value**
6-9 years	1 (0.3)	187 (99.0)	**188**	3.9	0.046
≥ 10 years	7 (3.3)	205 (96.7)	**212**		
**Seborrheic dermatitis**					
Age-groups	**positive (%)**	**negative(%)**	**total n = 400**	**x** ^**2**^	**p-value**
6-9 years	0 (0.0)	188 (100.0)	**188**	4.49	0.05
≥ 10 years	5 (2.4)	207 (97.6)	**212**		
**Scabies**					
Age-groups	**positive (%)**	**negative (%)**	**total n = 400**	**x** ^**2**^	**p-value**
6-9 years	29 (15.4)	159 (84.6)	**188**	9.16	0.002
≥ 10 years	13 (6.1)	209 (93.9)	**212**		
**Pediculosis**					
Age-groups	**positive (%)**	**negative (%)**	**total n = 400**	**x** ^**2**^	**p-value**
6-9 years	8 (4.3)	180 (95.7)	**188**		
≥ 10 years	0 (0.0)	212 (100.0)	**212**		
**Pyodermas**					
Age-groups	**positive (%)**	**negative (%)**	**total n = 400**	**x** ^**2**^	**p-value**
6-9 years	33 (17.6)	155 (82.4)	**188**	10.36	0.003
≥ 10 years	15 (7.1)	197 (92.9)	**212**		

**Prevalence of infectious dermatoses**: two hundred and eighty nine (72.3%) of the 400 pupils had infectious dermatoses; while 111 (27.7%) had no infectious dermatosis. The five most prevalent clinical forms of infectious dermatoses, in order of decreasing frequency, were Tinea capitis (35.2%), scabies (10.5%), Tinea corporis (5.8%), Tinea pedis (5.5%), and impetigo (5.0%). Some respondents had multiple clinical forms with a prevalence of 13% (Table 3). When the cases were grouped according to their presumed aetiologies, most of the infectious dermatoses were of fungal type, which was present in 213(73.7%) of the 289 affected respondents. Parasitic dermatoses, pyodermas and viral dermatoses followed in that order, with respective frequencies of 59 (16.6%), 48 (20.4%) and 24(8.3%). Among those 213 respondents who had fungal dermatoses, 141 (66.2%) had Tinea capitis; 23 (10.8%) had Tinea corporis; 22(10.3%) Tinea pedis; while 11 (5.1%) had onychomycosis. Eight pupils (3.8%) had Pityriasis versicolor. A similar proportion had seborrheic dermatitis (3.8%). The clinical forms of viral dermatoses seen among the respondents were: Orolabial herpes, 33.3%; Measles, 33.3%; Chicken Pox, 20.8%; and cutaneous warts, 12.6%. Scabies constituted the main parasitic dermatosis (71.2%). Others, in this category, included pediculosis (15.3%). Cases of linear dermatitis (reported to be caused by a crawling arthropod) constituted 13.5% of the parasitic dermatoses. Most (41.7%) of the bacterial dermatoses presented clinically as impetigo. Other observed clinical forms were folliculitis, 25.0%; furuncles, 22.9%; and carbuncles, 10.4%.

**Table 3:** Prevalence of major clinical forms of infectious dermatoses

Clinical forms	Frequency (n = 400)	Percent (%)
**Tinea capitis**		
Yes	141	35.2
No	259	64.8
Scabies		
Yes	42	10.5
No	358	89.5
**Tinea corporis**		
Yes	23	5.8
No	377	94.2
**Tinea pedis (athlete's foot)**		
Yes	22	5.5
No	378	94.5
Impetigo		
Yes	20	5.0
No	380	95.0
**Multiple clinical forms**		
Yes	52	13
No	348	87

**Age and sex specific prevalence**: while the overall prevalence infectious dermatoses was significantly more among the males than among the females(80.4% vs 67.2%), specific types of infectious dermatoses were significantly more prevalent among the females. This was most obvious in cases of Pityriasis versicolor and Pediculosis in which only females were affected. (Tables 1) The prevalence of infectious dermatoses rose from age 64.4% among the 6 -7 age group and peaked at 77.4% among those aged 8-9 years. With further increasing age, the prevalence decreased gradually through 77.1% (among those aged 10-11) to 67.6% (among those aged 12). The overall association between prevalence of infectious dermatoses and age was, however, statistically significant. (p = 0.007) When the ages of the pupils are categorized into “6-9 years” and “10 or more years”, Tinea capitis, Tinea corporis, Pityriasis versicolor, Seborrheic dermatitis and Pediculosis were found to be significantly more prevalent among those aged ≥10 years. All the cases of Seborrheic dermatitis and Tinea corporis occurred only in the ≥10 age-group. Pyodermas and scabies were significantly more prevalent in the 6-9 year age-group; and all cases of Pediculosis occurred in this age-group (Table 2).

## Discussion

The study population consisted of 400 children made up of 153 males and 247 females. This male/female ratio of 1: 1.5 indicates a higher school enrollment among females. The low school enrollment of males is attributable to the greater concern of the boys and their parents for making money. So there is an early diversion of the attention of the boys towards learning a trade. The greater encouragement of female education is significant, with respect to Millennium Development Goals ‘goals 2 and 3’ [33]. There is need to encourage female as well as male education to improve the literacy level of society for social and economic development. The mean age of the children was 9.43 ± 2.35; and majority of the children were of age 10-12. The ages of the children were within the reference school-age range of 6-12 years [34]. The prevalence of infectious dermatoses was found to be 72.3%. This value is above the 40.4% and 49.5% reported among primary school children in South western Nigeria [18, 28]; and is close to the 80% reported among children in Ethiopia [25]. The high prevalence, such as was found in this study, could therefore be a reflection of severely defective environmental health conditions that was obvious in this study population. This finding corroborates the earlier report that the high prevalence of infectious dermatoses is an index of socioeconomic development [35]. All the markers of low socioeconomic conditions were present in the study community [36, 37]. These include low level of education, low socioeconomic status of individual families, poor personal hygiene practices, inadequate environmental health practices, subsistence agrarian occupations, subsistence artisan occupations, petty trading, low access to potable water and adverse socio-cultural practices [36, 37]. All these point to the fact that the system of governance at the Local governments level leaves much to be desired and needs to be improved upon. Multiple skin lesions were found among 13.0% of the pupils. This compares with the 13.8% reported in Shagamu, Nigeria [38]; and contrasts with the 6% and 3.7% reported in India [39] and Romania [40] respectively. Generally, the prevalence of multiple infections indicate high transmission rate and the prevalence in this study is as expected, because similar factors-including poor hygiene, sordid environments and inadequate water supply-to enhance the transmission efficiency of infectious dermatoses pathogens [40]. The four aetiologic classes of infectious dermatoses were seen among the primary school pupils in this study. The commonest was fungal dermatoses, which affected 73.7% of those who had infectious dermatoses. The other types of infectious dermatoses included parasitic, bacterial and viral, with 20.4%, 16.6% and 8.3% respectively. The preponderance of fungal dermatoses in this study agrees with the findings of previous studies in Nigeria [18] and Tanzania [3]. Reasons that have been adduced for such preponderance of fungal dermatoses include the tropical climate (which predisposes to excessive sweating), poor hygiene, dirty environment, overcrowding, intimate association with animals, and scarcity of water [39], which were observed in the study environment. Moreover, the high transmission-efficiency of cutaneous mycoses can be related to the fact that the disease is usually strongly rooted in the family and that there is a high prevalence of healthy carriers [35, 39]. Treated cases and untreated cases are all capable of transmiting fungal dermatoses, as exfoliated corneal cells and hairs remain infective for long, thereby enabling transmission by fomites [40, 41]. Furthermore, there is the existence of dermatophytosis “reservoirs” in unhygienic nails [42, 43]. Although the spectra of skin infections seen in this study was generally similar to those reported studies in Nigeria [18, 28], there were some differences in the pattern of viral and parasitic dermatoses. A report from Ijesa-land, Nigeria, in addition to the other viral dermatoses found in this study, showed cases of molluscum contagiosium [28]; while no viral dermatosis was reported from Ibadan, Nigeria [18]. The finding of children with chicken pox and measles rashes, in this study, could possibly be a consequence of the inadequate immunization coverage for the children in early childhood. Also important is the fact that the school health programme is non-existent or ineffective and needs to be improved upon. None of the authors who studied prevalence of infectious dermatoses among Nigerian children found linear dermatitis [18, 28]. However, there was a case report in Western Nigeria [44]. The dermatitis, in that case report, was caused by body fluids of a crawling insect (Rove beetle) when killed by crushing against the skin [44]. Victims of this dermatosis, in this study, also associated cases with a certain crawling insect known locally as “otagburu nne omogo”.

Sex and age were assessed to determine their influence on the prevalence of infectious dermatoses. Sex was found to be a statistically significant determinant of the prevalence of infectious dermatoses. More males than females had infectious dermatoses with 80.4% and 67.2% respectively. This higher male sex prevalence agrees with reports from Western Nigeria [28, 38]. This finding could be due to the fact that females are more conscious of their appearances; and, as a result, they care more about personal hygiene, which promote health, than males. All the cases of pediculosis and pityriasis versicolor occurred only among females. This finding among the females corroborates other reports [27, 45]; and has been attributed to the tendency of females to wear long/grown hairs, share clothings/head-ties and wash their hairs infrequently. Control efforts on pediculosis and pityriasis versicolor should therefore be focused on the female sex. In this study, age was found to be a statistically significant factor in the overall prevalence of infectious dermatoses. This agrees with a report from Tanzania which showed that “age less than 10” was a predictor of infectious skin diseases among primary school children [3]. On the contrary, studies in Sagamu, Nigeria and Saudi Arabia did not report age as a statistically significant factor [38, 46]. Although the study from Sagamu did not generally find age a statistically significant factor, the prevalence of infectious dermatoses was observed to be highest among those aged 8 -9 years [38]. A similar observation was made in this study. The prevalence of infectious dermatoses peaked at 77.4% among those aged 8 -9 years and was least (66.4%) among those aged 6 -7 years. The higher prevalence of infectious dermatoses in the 8-9 age group, relative to the 6 -7 age group could be attributed to the waning of parental supervision of children's hygiene that occurs a few years after children have entered primary schools. It is obvious that at this young age the pupils need supervision especially in area of personal hygiene practices to reduce the prevalence of infections. It is therefore recommended that the parents should endeavour to supervise the bathing of the children. In this study, the prevalence of pyodermas, scabies and pediculosis were most prevalent among children less than 10 years. This observation further underscores the need for parental intervention in the hygiene practices of these very young children. Meanwhile, Tinea corporis, Pityriasis versicolor and Seborrheic dermatitis were more prevalent among children above 10 years. This finding could be attributed to the fact that as children mature the sebaceous glands accumulate and tend to enhance the growth of fungal agents responsible for these diseases [47–49]. Generally, the water shortage in the school and in the general community could be the reason for the high prevalence of infectious dermatoses. Therefore the local government should consider the provision of water for the schools and the community, a priority to improve the health status of the pupils in particular and the community in general.

## Conclusion

A wide clinical spectrum of infectious dermatoses were highly prevalent among the surveyed population. Sex and age were significantly associated with specific clinical types. Implementation strategies of school health programmes in similar rural communities should target these skin infections with due consideration of their specific sex- and age-correlates.
